# Long-Term Costs and Health Impact of Continued Global Fund Support for Antiretroviral Therapy

**DOI:** 10.1371/journal.pone.0021048

**Published:** 2011-06-23

**Authors:** John Stover, Eline L. Korenromp, Matthew Blakley, Ryuichi Komatsu, Kirsi Viisainen, Lori Bollinger, Rifat Atun

**Affiliations:** 1 Futures Institute, Glastonbury, Connecticut, United States of America; 2 Global Fund to Fight AIDS, TB and Malaria, Geneva, Switzerland; 3 Department of Public Health, Erasmus MC, University Medical Center Rotterdam, Rotterdam, The Netherlands; 4 Imperial College London, London, United Kingdom; McGill University, Canada

## Abstract

**Background:**

By the end of 2011 Global Fund investments will be supporting 3.5 million people on antiretroviral therapy (ART) in 104 low- and middle-income countries. We estimated the cost and health impact of continuing treatment for these patients through 2020.

**Methods and Findings:**

Survival on first-line and second-line ART regimens is estimated based on annual retention rates reported by national AIDS programs. Costs per patient-year were calculated from country-reported ARV procurement prices, and expenditures on laboratory tests, health care utilization and end-of-life care from in-depth costing studies. Of the 3.5 million ART patients in 2011, 2.3 million will still need treatment in 2020. The annual cost of maintaining ART falls from $1.9 billion in 2011 to $1.7 billion in 2020, as a result of a declining number of surviving patients partially offset by increasing costs as more patients migrate to second-line therapy. The Global Fund is expected to continue being a major contributor to meeting this financial need, alongside other international funders and domestic resources. Costs would be $150 million less in 2020 with an annual 5% decline in first-line ARV prices and $150–370 million less with a 5%–12% annual decline in second-line prices, but $200 million higher in 2020 with phase out of stavudine (d4T), or $200 million higher with increased migration to second-line regimens expected if all countries routinely adopted viral load monitoring. Deaths postponed by ART correspond to 830,000 life-years saved in 2011, increasing to around 2.3 million life-years every year between 2015 and 2020.

**Conclusions:**

Annual patient-level direct costs of supporting a patient cohort remain fairly stable over 2011–2020, if current antiretroviral prices and delivery costs are maintained. Second-line antiretroviral prices are a major cost driver, underscoring the importance of investing in treatment quality to improve retention on first-line regimens.

## Introduction

As of the end of 2009, over 5 million people were receiving antiretroviral treatment (ART) globally [1]; 2.5 million people through programs co-financed by the Global Fund to Fight AIDS, Tuberculosis and Malaria (Global Fund) [3]. Seventy-seven percent of Global Fund-supported patients were in sub-Saharan Africa, 15% in Asia, 3% in Latin America/Carribean, 3% in Eastern Europe & Central Asia each, and1% in the Middle East. By 2011, Global Fund-supported programs in 104 low- and middle-income countries will aim to scale-up to have 3.5 million people on ART according to service delivery targets of existing grants and approved proposals [4] — a remarkable success, given that in 2003 only 400,000 people with HIV in low- and middle-income countries were receiving ART [2].

Increased international financing has enabled the rapid growth in the number of people receiving ART, though including all those who do not currently have access to treatment will require US$ 7 billion annually, as a minimum estimate based on the WHO's 2006 treatment eligibility criteria [5]. While studies have projected increased financing needs to treat all those who need it [6], none have estimated the level of financing required to support the patients currently receiving ART, which could be seen as a minimum to funding requirements.

We use new data on Global Fund financing of ART programs to estimate the future funding required to enable the cohort of 3.5 million persons expected to be receiving ART in Global Fund-supported programs as of 2011 to continue receiving treatment through 2020. We also compute the health impact of continued provision of ART for this cohort, in terms of deaths averted/postponed and life-years saved, and explore key determinants of cost.

## Methods

We estimate the long-term costs and impact of Global Fund support by following the cohort of patients on treatment in 2011. New patients who will start treatment in Global Fund-supported programs over next years are not considered in the current analysis, for simplicity and in order to establish a minimum commitment. We estimate the future cost and health impact by projecting the number of patients that will still be alive and receiving treatment in future years by subtracting those that die of AIDS or non-AIDS causes. Although some patients may stop therapy voluntarily that does not reduce the obligation to continue them on treatment if they desire it.

We have used as a starting point the number of patients on ART with Global Fund support in 2011. While the actual grant delivery results for 2011 will not be available until early 2012, the funding committed for ART treatment is already in place, and supported programs have each set their target for the number of people to receive treatment through ongoing grants and approved grant proposals for next calendar years.

Patients are assumed to survive according to recent, internationally agreed best estimates of current rates of survival, as discussed below. Patients may also fail first line treatment and require more expensive second line. Although many patients live in countries where the provision of second line is inadequate we assume that all patients get second line when they need it, for the purpose of estimating the funds required to provide necessary treatment.

Impact is measured in terms of deaths averted and life-years gained, as detailed below, comparing the actual patient cohort with a counterfactual scenario in which essentially all those patients on treatment in 2011 would die within a few years if they could not longer access treatment.

### Cohort survival on first-line and second-line treatment

For the 2011 cohort of 3.5 million patients, ART need in future years is estimated based on the retention and survival of those patients in each subsequent year. According to reports submitted to the World Health Organization (WHO) by 38 national AIDS programs in low- and middle-income countries in 2008, the proportion of patients (adults and children) remaining on treatment – i.e. not dying and not lost to follow-up – averaged 80% at 12 months after treatment initiation, and 75% after 24 months, 74% after 36 months and 73% after 48 months [2]. These figures vary by region with the best 48-month retention rates achieved in Latin America and the Caribbean (74%), followed by North Africa and the Middle East (78%). Lower retention rates are achieved in Europe and Central Asia (67%), sub-Saharan Africa (67%) and East, South and South-east Asia (55%). Based on an unweighted average across the regional survival/retention data, we assume an annual survival of 79.5% for the first year, and 96% survival for each subsequent year, for all countries. For example, in 2011 alone there are 790,000 new patients who initiated treatment, 79.5% of whom will survive into 2012, while 96% of continuing patients will survive to 2012. After 2012 all patients continue with a 96% survival rate.

For the start year of projections, 2009, we used the WHO 2008 survey for 38 National AIDS Programs to determine country-specific proportions of patients on second-line regimens. For countries not participating in this survey, we applied regional weighted average proportions. Across all Global Fund-supported countries, the weighted average proportion was 2.5% of patients on second-line regimens. For subsequent years, we calculated the proportion of patients on second-line regimens using regional migration rates with an average of 1.9% of patients who survived on first-line regimens switching to second-line regimens each year [7].

### Health impact

We estimated the health benefits of Global Fund-supported ART by comparing survival of patients initiating and continuing treatment, with a counterfactual cohort for which continued treatment was not available from 2011 onwards.

To calculate the health impact of continued ART, we assumed that all HIV-infected people starting ART have advanced disease and meet standard ART eligibility criteria, according to the WHO's 2006 Treatment Guidelines which propose ART for HIV-infected people with clinical stage III or IV disease, and/or CD4 below 200/µL [8]. This is in line with program data reported by low- and middle-income countries to the 2008 WHO survey, in which CD4 counts at treatment initiation are often well below 200/µL, for example a median of 67/µL among 13 African countries [2]. Survival on ART is calculated as specified above. For the counterfactual scenario that these patients in need would not continue to receive ART, we calculated mortality based on data on people in acute need of treatment but never on treatment. Based on the time from infection to AIDS deaths as analyzed by the ALPHA network, a collaboration of cohort studies in Africa [9], [10], the cumulative proportion of a cohort currently on ART that would die if ART were stopped would be 18% after 1 year, 46% after two years, 64% after three years, 76% after 4 years, 84% after 5 years, and 97% after 5 years – with median survival being 3 years. Using these cumulative mortality rates in a Weibull survival curve [12], we calculated the number of people that would still be alive over subsequent calendar years with and without ART, to estimate numbers of deaths averted and life-years saved in each calendar year. Although most patients on treatment in 2011 started treatment in earlier years and thus may have improved immune systems, we assumed that if treatment were stopped abruptly they would have survival patterns similar to those who became eligible for treatment but never received it, so survive for a median slightly more than 2 years after the cessation of treatment.

### Cost per patient-year of ART

Estimates of cost per patient-year of ART are shown in Table 1. These costs represent overall, recurrent patient-level direct costs, to which the Global Fund contributes alongside other partners and domestic resources.

**Table 1:** Assumed costs of ART delivery.

Cost component	US $	Source
First-line ARVs: 1 patient-year	204#	Global Fund Price & Quality Reporting system and WHO Global Price Reporting mechanism [4], [5], for the patient-weighted mix of the 6 first-line or second-line ARV regimens that were most commonly used in low- and middle-income countries over 2006–8 [1].
Second-line ARVs: 1 patient-year	1,238#	
Laboratory testing: 1 patient-year	180	Comprehensive costing studies (see Supporting Online Material, Table S1)
Treatment delivery: 1 patient-year	138	WHO-CHOICE country estimates [6] (see Supporting Online Material, Table S2)
End-of-life treatment of opportunistic infections: per patient lifetime	160	During a patient's *last* year on ARV therapy only. Based on WHO-CHOICE [6] and literature review of non-ARV therapy costs of HIV care. [See supporting on-line material]
**Total first-line ART (1 patient-year)**	**487** #	
**Total second-line ART (1 patient-year)**	**1,521** #	

# ARV and ART cost assumptions are based on country-specific estimates of ARV and service delivery cost and a fixed cost for laboratory and end-of-life treatment of opportunistic infections. The shown average ‘Total’ is weighted across Global Fund-supported AIDS programs according to their number of patients at end-2009. The shown weighted average costs of the individual components do not add up to the weighted average total cost per patient, because of considerable variation among countries in the cost of ARVs and treatment delivery.

Prices for both first-line and second-line ARVs vary substantially by country but have declined over recent years. Based on country-reported procurements reported through the Global Fund's Price and Quality Reporting system and WHO's Global Price Reporting Mechanism, median prices across Global Fund-supported countries were $204 for first-line drugs and $1238 for second-line drugs as of 2009 [4].

In most programs ART patients are monitored with laboratory tests for CD4 counts, blood chemistry and hematology. Some programs also include viral load monitoring. Treatment protocols usually specify monitoring every three to six months. Our review of 15 published reports from low- and middle-income countries on the costs of laboratory tests found a median annual cost of $180 for monitoring each patient receiving ART (Table S1) [13]–[27].

Service delivery costs for ART care (personnel, supplies, facilities, etc.) also vary by country; depending on the frequency of visits, the type and level of health care facilities and clinical staff used for monitoring patients and local health worker salaries. Our review of studies from eight countries which have reported on the annual number of in-patient days and out-patient visits estimated a median of 9.5 out-patient visits and 1.6 in-patient days per ART patient per year (Table S2) [7]–[9], [11], [12], [28]–[30]. Country-specific estimates of the cost per in-patient day in a primary-level hospital and a 20-minute out-patient visit are available from the WHO CHOICE database [31]. We combined global average frequencies of in-patient days and out-patient visits with country-specific costs from WHO-CHOICE to estimate a median annual cost of $138 per patient receiving ART.

Using review results we calculated non-ART care requirements, once HIV patients fail ART, at 5.5 out-patient visits, 9.7 in-patient days and $49 in non-ARV drug costs per patient-year. Based on WHO-CHOICE country estimates of the cost of hospital days and outpatient visits in each country [32], this corresponds to a median health care cost of US$ 320 per year. For patients dying on ART We added end-of-life care costs of US$ 160 per patient, incurred during the last half year before death from HIV/AIDS.

All costs are expressed in 2008 US dollars.

### Sensitivity analysis: ARV price, cost and regimen changes

Cost estimates were repeated in sensitivity analysis that varied, univariately, the future prices of first-line and second-line ARVs, the mix of ARV regimens used and the rate of migration from first-line to second-line ARV regimens. (In our model, we assumed these changes to not affect health impact.)

## Results

### Survival and cost

**Figure 1:**
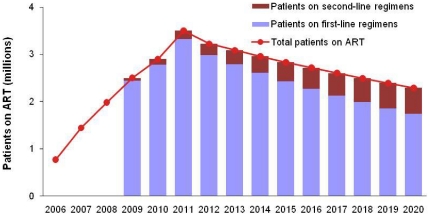
HIV/AIDS patients on ART in Global Fund supported programs according to end-2010 grant results and 2011 grant service delivery targets of ongoing grants and approved grant proposals (up to and including Round 10), and expected retention on first-line (FL) and second-line (SL) ARV regimens over future years.

**Figure 2:**
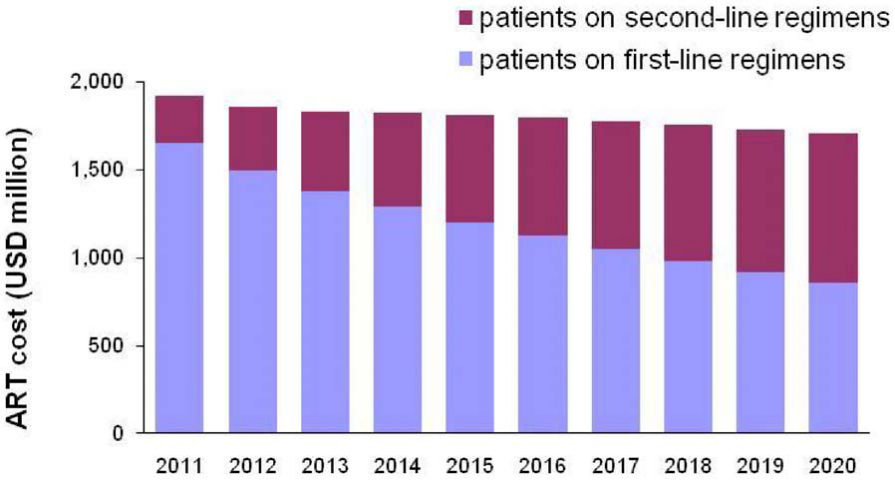
Cost of continued ART for Global Fund-supported patients as of 2011: first-line versus second-line regimens.

Of the 3.5 million people receiving ART with Global Fund support in 2011, we estimate that 2.3 million will still be alive and receiving treatment in 2020. (Figure 1) The proportion of these patients receiving second-line regimens will rise from 2.5% in 2009 to 24% by 2020. The full annual cost of supporting these patients will decline by 10% from about US$ 1.9 billion in 2011 to US$ 1.7 billion in 2020. Although the number of people remaining on treatment will decline by 35% by 2020, the larger proportion on more expensive second-line treatment means annual drug costs will decline by only 17%. By 2020, the 24% of patients on second-line regimens will account for 50% of the total program cost (Figure 2).

### Health impact

**Figure 3:**
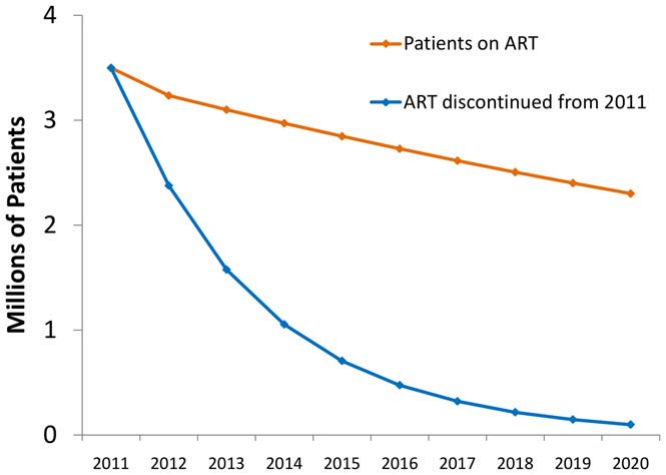
Number of patients on ART and patients surviving if ART were discontinued from 2011.

The health impact of continuing the 2011 cohort of patients on ART will be quite large since all those receiving ART today would die by 2020 if they stopped ART. The largest number of deaths (860,000) is averted in 2012, the year after the 2011 cohort reaches its full size (Figure 1). The annual number of deaths averted then falls gradually over subsequent years (Figure 3). From 2018 onwards, no additional deaths are averted, since most of those people currently on ART would be dead by then if they stopped treatment in 2010. Continuation of the 2011 cohort of patients on ART will postpone some deaths until 2018–2020, leading to more deaths in those years with continued ART than without.

**Figure 4:**
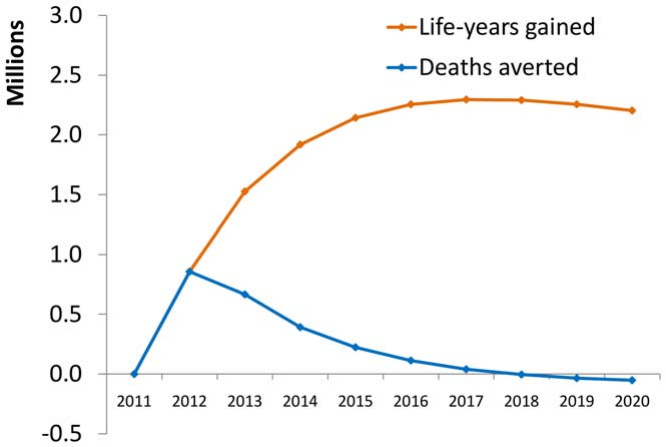
Expected health impact: mortality and lives saved from ART: Deaths averted and life-years saved.

The averted deaths correspond to 860,000 life-years saved in 2012, increasing to 2.3 million annual life-years saved by 2017 (Figure 4). Cumulatively over 2011–2020, continued ART is estimated to save 17.7 million life-years.

### Sensitivity analysis: ARV price, cost and regimen changes

**Figure 5:**
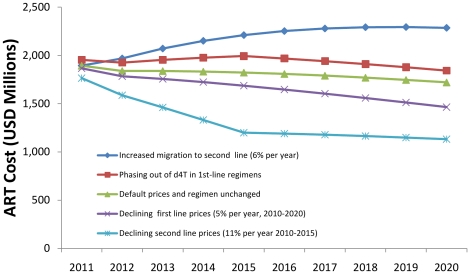
Effect of changing ARV regimens and prices on ART cost.

The future investment required to support those currently receiving ART will be influenced by a number of factors including changes in ARV prices, the distribution of patients over ARV regimens of differing prices, and patient laboratory monitoring practices. The median prices of the most commonly used first-line ARVs declined by an average 12% per year from 2006 to 2009 [3]. This pace of decline is not likely to continue for first-line drugs. In 2010, the Global Fund committed to achieve a median 5% annual price decline for commonly used first-line ARVs across the ART programs it supports, as a corporate Key Performance Indicator [33]. For the 2011 cohort, a 5% annual decline after 2010 would reduce the estimated cost in 2020 by $260 million or by 15% compared to our baseline cost estimate for 2011 of $1.7 billion (Figure 5).

Considering the higher current prices there is more scope for faster declines in second-line ARV prices in coming years [34]. An annual reduction in second-line prices of 11% every year until 2015, with prices maintained at 2015 level thereafter, in line with what has been achieved over 2006–2009 and with the Global Fund Key Performance Indicator for first-line ARVs, would diminish estimated costs in 2020 by $560 million or by 34%.

The average per-patient cost of first-line regimens may rise in the future as countries implement the revised 2009–10 WHO ART guidelines [34] to phase out the use of stavudine (d4T), the least expensive first-line drug. If all patients currently on d4T-containing regimens were switched to other first-line regimens in proportion to 2008 usage patterns [1], the median price of first-line ARVs would rise from $204 in 2009 to $254 in 2015 per patient per year. The financing needs in 2020 would consequently increase by at least $120 million (7%) in total. (The increase could be even more if the switch to non-d4T-containing regimens leads to better adherence and survival on ART.)

Countries that use routine viral load monitoring have higher migration to second-line regimens, around 6%, than those that do not routinely monitor viral load (2.6% in sub-Saharan Africa and Latin America and the Caribbean, and 1.1% in Asia). If all Global Fund-supported countries had 6% annual migration to second-line regimens, then financing needs in 2020 would increase by $560 million, a 33% increase.

## Discussion

The number of people receiving ART in low- and middle-income countries has expanded rapidly in recent years. Much work has focused appropriately on what is required to continue this scale-up to reach all those in need of treatment. This study examines a previously unexplored aspect of treatment, the financing required to continue supporting patients receiving ART.

Investments by the Global Fund together with other partners have enabled and catalyzed a rapid expansion of HIV programs in low- and middle-income countries that is reducing mortality and new infections [36], [37]. As of the end of 2009, Global Fund grants co-supported, fully or partially, around half of all patients receiving ART globally. For the 3.5 million patients receiving ART as of 2011, at current cost levels the annual resource need ranges from US$ 1.9 billion in 2011 to US$ 1.7 billion in 2020. The Global Fund does not support all of these costs; the USA President's Emergency Plan for AIDS Relief (PEPFAR), other donors, national governments and other domestic resources also provide partial support for the patients benefiting from Global Fund investments. Based on expenditure reporting from Global Fund recipient HIV/AIDS programs, across countries with Global Fund-supported ART the average annual expenditure from Global Fund grants per person-year of ART was estimated at about a quarter of the full program-level ART delivery costs estimated in this paper [4]. This is roughly consistent with the Global Fund's share in overall international AIDS control funding, which in 2008 equalled 19% [38].

The costs of treatment in this analysis vary only by first and second line treatment. In reality costs may be different for different types of patients, pregnant women, children, TB patients. We have used an average per patient cost, which implicitly assumes that the distribution of patients by type will not change.

Sensitivity analyses showed that program costs are sensitive to ARV prices and regimens used, in particular for second-line ARV regimens. A balanced use of lower-cost generic ARVs and more expensive innovator drugs will impact costs. Efforts to improve treatment quality to retain patients on first-line regimens will be critically important to manage future costs. Equally, considerable variations in the protocols and frequencies of laboratory testing and associated cost point to opportunities for efficiency gains in patient monitoring. Recent studies in resource-constrained settings suggest that CD4-based patient monitoring is generally cost-effective compared to clinical monitoring alone, but that viral load (HIV RNA) monitoring is not always cost-effective [45]–[49]. While lack of laboratory capacity should not impede countries to roll-out first-line ART, there is a need to develop affordable, point-of-care viral load and CD4 assays that can supplement clinical monitoring and ensure timely detection of treatment failure and regimen switches [50].

To further refine this type of assessment, we will need progressively better information on country-level variation and determinants of cost of ART delivery [39]. Recently established routine tracking of national program budgets and expenditures can contribute to fill this current data gap [40], and inform the effects of ARV drug price declines, changing treatment eligibility criteria and regimen mixes, and the proportion of patients on first- and second-line ART [2], [9]. As of now, a paucity of country-level expenditure data on especially non-ARV components limits our understanding of current and future ART delivery cost in different settings. Routine expenditure data collection by national AIDS programs will also assist in optimizing value for money, an important determinant of future national and international (donor) commitments to ongoing ART scale-up [4], [6], [51]–[54].

Also, program achievements in terms of patient retention and survival, on first-line and second-line regimens, deserves improved monitoring and assessment. In-depth studies and evaluations in countries with relatively advanced patient tracking systems suggest that routine national program records may overestimate actual retention and survival in several high-HIV countries, given elevated mortality among patients lost-to-follow-up, and (passive) over-counting of retention time among patients who dropped out from recent clinic or pharmacy appointments. On the other hand, patients transferring between facilities may be over counted as new initiations and undercounted in retention [55]–[58]. In high-HIV countries with substantive ART coverage, triangulation of program and clinical data with population-level mortality trends from vital registration, surveys and censuses would provide the ultimate reassurance on population-level health impact [59]–[60].

We estimate continued investment needs for a cohort of 3.5 million people receiving ARVs. The global need for treatment and financing of HIV is far larger than this number. The 2010 revised WHO HIV Treatment Guidelines [35] raise the estimate of the number of people in need of treatment by about 50% [41]. UNAIDS estimates suggest funding for ART would have to expand to US$ 7 billion annually by 2015 in order to achieve 80% coverage of those most acutely in need (with CD4 counts under 200 cells/µL), and would further increase by 50% to achieve 80% coverage under the expanded treatment eligibility criteria (covering all those with CD4 counts under 350 cells/µL) [41]. Globally the number of patients on ART has been increasing at almost one million patients per year between 2006 and 2008. If that rate of scale up were to continue through 2020 and the Global Fund continued to support about 62% of ART patients as it did in 2009 [3], then the number of patients needing Global Fund support would increase to 9 million by 2020. This would correspond to an annual co-investment need by the Global Fund of $5.2 billion—assuming constant grant expenditures per patient supported and continued proportionate co-investments from domestic sources, PEPFAR and other donors. However, there is no guarantee that scale-up in funding from domestic and other donor sources would keep pace with the ongoing rate of new ART patient initiations. Thus the Global Fund is likely to remain an important source of support for most countries. While some middle-income countries with strong economic growth could assume greater responsibility for funding their own programs, thus reducing the Global Fund investment needs, this is unlikely to be the case in low-income countries where the majority of Global Fund beneficiaries live [6].

While this study examined ART costs for existing patients through the year 2020, investment needs continue beyond this date, as by that year 2.3 million people (66% of the 2011 cohort) will still be alive and in need of continuing treatment Support for these patients will also include non-ART costs such as prevention and treatment of opportunistic infections including tuberculosis and malaria [61], [62]. Costs considered did not include the above-facility level costs such as human resources at drug distribution centers, district/provincial/national program management, monitoring and evaluation, health worker training in AIDS management and health system strengthening in general. A recent evaluation across 6 PEPFAR-supported African AIDS programs estimated above-facility-level costs at 20% of overall costs [63].

In conclusion, the rapid expansion of ART in low- and middle-income countries in recent years has produced dramatic declines in mortality and represents an important success in our efforts to improve global health. Sustaining and protecting gains will require sustained investment from international donors such as the Global Fund, as well as sustained contributions by the governments of supported country programs.

## Supporting Information

Table S1 **Laboratory costs of ART, USD per patient-year.**
(DOCX)

Table S2 **Service delivery costs of ART, per patient-year.**
(DOCX)
