# Supplementary material for: Long-Term Costs and Health Impact of Continued Global Fund Support for Antiretroviral Therapy
Source: PLoS One. 2011 Jun 23;6(6):e21048. doi: 10.1371/journal.pone.0021048 (PMC3121720; doi:10.1371/journal.pone.0021048)
Supplement: Table S1 — Laboratory costs of ART, USD per patient-year. (DOCX) [file pone.0021048.s001.docx]

| **Country** | **Comments** | **Cost** | **Source** |
| --- | --- | --- | --- |
| Cote d'Ivoire | CD4 every 6 months at $25, plus $7.91 for initiation of ARV - averaged over four years | $52 | [7] |
| Ethiopia |  | $207 | [14] |
| Mexico | Average of 1st, 2nd and 3rd year after ART initiation; | $366 | [9] |
| Nigeria | Assume monthly visits for ARV patients | $204 | [10] |
| Thailand | ALL MODEL ASSUMPTIONS: ARV patients have monthly OPV, laboratory tests 4x/yr, AIDS patients without ARV average length of stay=6.4 & average hospital admissions=3 | $459 | [11] |
| Uganda | 12 ARV visits per year - no costing of IPD; | $74 | [12] |
| Zambia | 1 session to initiate ARV, 4 sessions per year to monitor | $178 | [13] |
| South Africa | Actual tests in patient pop: 1.8 CD4 tests/year (at R60/test), 1.6 Viral Load tests/year (at R300/test), 5 ALT (R36), 5 FBC (R46), and 2 chemistry (R24-53), converted to USD (2004 rate R 6.46) (Laboratory tests 13% of total costs) | $156 | [42] |
| Brazil | Reports that Brazilian Health Ministry intends to do 400,000 tests at $18 million, assuming 4 tests/year | $180 | [14] |
| Caribbean | 2 CD4 and viral load tests per patient per year. | $400 | [15] |
| Haiti | Total reported is $130 for a mean number of 11.3 ART monitoring laboratory tests; 1.3 CD4 and 0.4 chest radiographs per 299 days of treatment. ((Scaled up to 365 days of treatment brings cost to $158.70.)) (Laboratory tests comprised 15% of total costs.) | $159 | [16,43] |
| South Africa | Includes drug toxicity and clinical efficacy assessments - weekly for first 4 weeks and monthly thereafter; CD4 and viral load tests - at baseline and every 2 months; and HIV genotypic resistance - at baseline and at 6 months (Laboratory costs 21% of 6-month total $1286) | $544 | [17] |
| South Africa (KwaZulu-Natal) | Laboratory cost is stated without details on what tests are included. ((Total average costs were R 6848, with breakdowns: personnel R1927, laboratory R1514 (US$223.63), equipment R44, supplies R90, drugs R3208, utilities R62. Converted to USD (2006 rate R 6.77))) (Laboratory costs were 22% of total.) | $224 | [44] |
| Rwanda | Monitoring laboratory tests per patient per year (55/yr), CD4 tests, 2 per year (10.52/each?) | $66 | [18] |
| South Africa | CD4 $ 9.32 per test, HIV RNA load $42.62 per test, and alanine transaminase $4.76 per test.  (Also reports cost per person-year in program, $92.26, at end of year one.) | $92 | [19] |
| **Median** |  | **$180** |  |

Abbreviations: ARV = antiretroviral, CD4 = CD4 cell count, OI = opportunistic infections, OPV = out-patient visit, OPD out-patient day IPD = in-patient day, R=South African Rand, ALT = alanine aminotransferase liver function test, FBC =full blood count.
