# Supplementary material for: Long-Term Costs and Health Impact of Continued Global Fund Support for Antiretroviral Therapy
Source: PLoS One. 2011 Jun 23;6(6):e21048. doi: 10.1371/journal.pone.0021048 (PMC3121720; doi:10.1371/journal.pone.0021048)
Supplement: Table S2 — Service delivery costs of ART, per patient-year. (DOCX) [file pone.0021048.s002.docx]

| **Country** | **Comments** | **Out-Patient Visits** | **In-Patient Days** | **Source** |
| --- | --- | --- | --- | --- |
| Ethiopia |  | 9 |  | [8] |
| Mexico | Average over 1st, 2^nd^ and 3rd year on ART | 10 | 12.17 | [20] |
| Nigeria | Assume monthly visits for ART patients | 12 |  | [10] |
| South Africa | Average over 1st, 2ndand 3rd year on ART:  , >3 year; OI =Average between CD4<50/μL and CD4 50-199/μL. | 5.62 | 0.45 | [21] |
| South Africa | Averaged Non-AIDS/AIDS usage for ART/No-ART patients; did not split drug costs out from service delivery for OI treatment | 8.17 | 1.56 | [22] |
| Uganda | 12 OPV visits per year per ART patient; no costing of IPD. | 12 |  | [12] |
| Zambia | 1 OPV to initiate ART, 4 OPV per year to monitor ART. | 4 |  | [13] |
| **Median** |  | **9.5** | **1.56** |  |

Abbreviations: ART = antiretroviral treatment, CD4 = CD4 cell count, IPD = in-patient day, OI = opportunistic infections, OPD =out-patient day, OPV = out-patient visit; IPD =in-patient day, R=South African Rand.
